# Measuring the Coefficient of Friction of a Small Floating Liquid Marble

**DOI:** 10.1038/srep38346

**Published:** 2016-12-02

**Authors:** Chin Hong Ooi, Anh Van Nguyen, Geoffrey M. Evans, Dzung Viet Dao, Nam-Trung Nguyen

**Affiliations:** 1 Queensland Micro- and Nanotechnology Centre, Griffith University, 170 Kessels Road, 4111 Queensland, Australia; 2 School of Chemical Engineering, University of Queensland, St Lucia, 4072 Queensland, Australia; 3 School of Engineering, University of Newcastle, Callaghan, NSW 2308, Australia

## Abstract

This paper investigates the friction coefficient of a moving liquid marble, a small liquid droplet coated with hydrophobic powder and floating on another liquid surface. A floating marble can easily move across water surface due to the low friction, allowing for the transport of aqueous solutions with minimal energy input. However, the motion of a floating marble has yet to be systematically characterised due to the lack of insight into key parameters such as the coefficient of friction between the floating marble and the carrier liquid. We measured the coefficient of friction of a small floating marble using a novel experimental setup that exploits the non-wetting properties of a liquid marble. A floating liquid marble pair containing a minute amount magnetite particles were immobilised and then released in a controlled manner using permanent magnets. The capillarity-driven motion was analysed to determine the coefficient of friction of the liquid marbles. The “capillary charge” model was used to fit the experimental results. We varied the marble content and carrier liquid to establish a relationship between the friction correction factor and the meniscus angle.

A liquid marble is a liquid droplet coated with hydrophobic powder, which can roll on a solid surface^1^,^2^,^3^,^4^,^5^ and float on a liquid surface due to its non-wetting coating^6^,^7^,^8^,^9^,^10^,^11^,^12^,^13^,^14^,^15^,^16^. A liquid marble can be driven across a liquid surface using thermocapillarity^17^,^18^. Liquid marbles served as micro bioreactors for culturing cells^19^,^20^,^21^,^22^,^23^. A number of excellent review papers provided a comprehensive summary on properties and applications of liquid marbles^1^,^14^,^24^,^25^,^26^. Recently, Bormashenko *et al*. demonstrated that a floating liquid marble can propel itself across the water surface due to the Marangoni solutocapillary effect^16^. Self-propulsion provides a means of transport for a small volume of an aqueous solution. Our previous study on the autonomous motion of a floating liquid marble focused on operation parameters such as the geometric constraint and the concentration of a volatile compound within the marble^13^. The dynamics of the motion was beyond the scope of the previous study as critical parameters such as the coefficient of friction of a floating marble were unknown. The marble motion is driven by surface tension gradient and resisted by friction. The knowledge of both the friction force and the resultant motion can lead to the determination of the surface tension gradient. This gradient can then be correlated to the amount of volatile compound released to the surface of the carrier liquid. Insights into the autonomous motion of a self-propelled floating liquid marble allows for better prediction of its velocity, carrying capacity and lifetime.

The coefficient of friction of a solid spherical particle straddling between two fluids have been studied extensively^27^,^28^,^29^. Some previous studies analysed the motion of small spherical particles under the action of known capillary forces^30^,^31^. The parameters were chosen such that the particles experienced Stokes drag. The Newtonian equation of motion was then applied to solve for the friction term, which has a correction factor. This model requires the measurement of several key parameters. First, the radius of the three phase contact line (TPCL) must be known to calculate the Stokes drag. Second, the meniscus angle created by the particle needs to be measured as it is related to the capillary force. The measurement of these two parameters for a solid spherical particle is relatively straightforward. However, a floating liquid marble deforms, and its meniscus angle does not necessarily depend on the apparent contact angle^12^. Furthermore, the radius of the TPCL varies with the marble volume. For simplicity, the liquid marbles investigated in this paper are sufficiently small to be assumed as a sphere.

To date, the measurement of the coefficient of Stokes friction for a floating liquid marble has not been reported. Although we have previously estimated the correction factor of the ideal Stokes friction coefficient based on indirect measurements and calculations^13^. In the present work we seek to measure this correction factor by applying known theories and a novel experimental setup, which exploits our recently reported magnetic actuation concept for liquid marbles^32^. A liquid marble can be controlled magnetically by filling it with a small amount of magnetite without significantly changing its density or surface tension. Two floating marbles containing magnetite were immobilised with permanent magnets positioned below. If the magnets are dropped vertically, the marbles attract each other through the capillary force of the deformed liquid surface. Using the calculated capillary force, we determined the correction factor for the coefficient of friction of the floating marbles. We then correlate this correction factor with the meniscus angle of the carrier liquid, thus creating a convenient method of estimating this factor.

## Theory

Two floating spherical particles, placed close to each other, experience a resultant force, 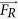. In the horizontal (or *x*) direction, the component of the resultant force *F*_*R*(*x*)_ acting on a particle is:


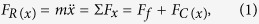


where *m* is the mass of the particle, 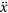 is the horizontal acceleration of the particle; and *F*_*C*(*x*)_ and *F*_*f*_ are the capillary and friction force, respectively. The friction force can be calculated using Stokes’ law^30^,^31^:


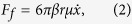


where *r* is the particle radius, *μ* is the dynamic viscosity of the carrier liquid, 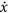 is the horizontal velocity of the particle, and *β* is the correction factor for the coefficient of friction.

**Figure 1:**
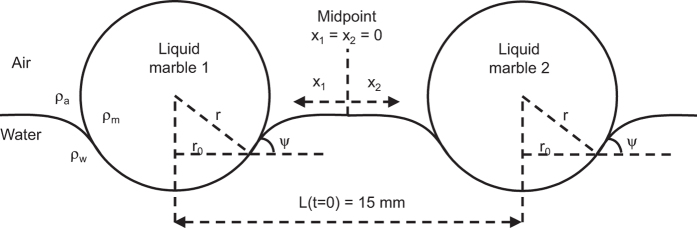
Schematic side view of a pair of floating liquid marbles. The coordinate system uses the midpoint as reference for the centroid positions of the two marbles. The distance *L* between the two marbles is set to 15 mm as one of the initial conditions.

The seminal work by Kralchevsky *et al*.^33^ concluded that each particle behaves like a “capillary charge”, which is analogous to electrically charged particles. The capillary forces can be expressed as:


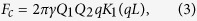


where *L* is the distance between the particles and the first order of the modified Bessel function of the second kind *K*_*1*_ that can be approximated as:


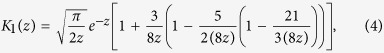


where *z* is a dimensionless quantity (*z* = *qL* in our case), *γ* the surface tension between the fluids, *q* the inverse capillary length of the carrier fluid:


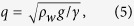


where *ρ*_*w*_ is the density of the carrier liquid. *Q*_*1*_ and *Q*_*2*_ are the capillary charges of individual particles that can be calculated as:


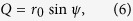


where *r*_*0*_ is the contact radius of the TPCL, which is typically much smaller than the distance between the two marbles *L* ref. 33. For example, in our experiments *r*_*0*_ is on the order of 5 × 10^−4^ m, whilst *L* is set to be 1.5 × 10^−2^ m. In Equation (6) is the meniscus angle of the carrier liquid relative to a reference horizontal level, Fig. 1. Assuming the marble remains spherical, the contact radius can be estimated as:


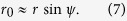


Substituting (7) into (6) results in:


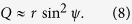


For two liquid marbles of the same composition and radius, *Q* = *Q*_1_ = *Q*_2_; *L* = 2*x* given that *x* is measured from the midpoint between the two marbles. Substituting *m* = (4π*r*^3^/3)*ρ*_*m*_, where *ρ*_*m*_ is the effective density of the liquid marbles, and making the appropriate arrangement in (1) gives:


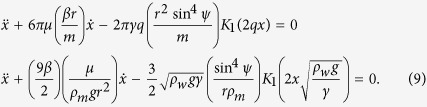


In (9), the physical properties (*μ, r, ρ*_*m*_ and *ρ*_*w*_) are known. From the experiments, *x* is measured as a function of time, and the resultant data can be differentiated with respect to time to obtain both velocity, 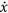, and acceleration, 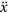. Consequently, the measurements can be used in combination with (9) to obtain the values of *β* for a known meniscus angle *ψ*.

The meniscus angle *ψ* is determined by application of a vertical force balance to the liquid marble (at equilibrium):


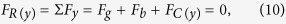


where *F*_*g*_ is the weight of the marble, *F*_*b*_ is the buoyancy force, and *F*_*C*(*y*)_ is the vertical component of the capillary force. Making the appropriate substitutions for *F*_*g*_, *F*_*b*_ and *F*_*C*(*y*)_ gives:


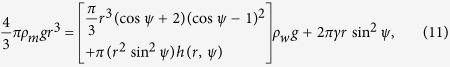


where *h*(*r, ψ*) is the height of the meniscus, measured vertically from the TPCL to the free liquid surface sufficiently far away from the marble; *h* is a function of *r* and *ψ*, and can be solved using methods described in our previous work^12^.

Equation (11) can be solved numerically to obtain an equilibrium value for *ψ* for a given set of values for *r, γ, ρ*_*m*_ and *ρ*_*w*_. With the value of meniscus angle *ψ* then, *β* can subsequently be obtained from (9). Note that the analysis given above is only valid for small particles, i.e. (*qr*)^2^ ≪ 1, and small meniscus slopes, i.e. 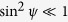
34,35. In our case, both (*qr*)^2^ and 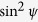 were on the order of 10^−1^.

The correction factor, *β* is known to be dependent on the amount of perturbation of the liquid interface, which in turn depends on the meniscus angle *ψ* and the submerged depth of the liquid marble. Since this depth is a function of *ψ* for a known volume, *β* should be a function of *ψ* as well. For a small floating liquid marble, the buoyancy force *F*_*b*_ is small compared to the surface tension force *F*_*C*(*y*)_^15^. Neglecting *F*_*b*_ in (10), Equation (11) can be simplified as:


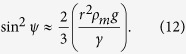


As *β* should be a function of *ψ* for a given volume, it should also be a function of the liquid surface tension, *γ* and *ρ*_*m*_. Note that the liquid density and marble surface tension terms are missing, because buoyancy force and marble deformation are neglected. This assumption gives us a convenient way to estimate *β* based on reasonably measurable quantities. In this paper, we vary the effective marble density *ρ*_*m*_ and the carrier liquid surface tension *γ* to determine the relationship between the correction factor *β* and sin *ψ*. The marble radius *r* is kept constant, whereas *ρ*_*m*_ is measured for marbles containing different NaCl concentrations. The surface tension *γ* and the density *ρ*_*m*_ were changed in our experiments, independently.

With an aspect ratio of ε ≈ 0.9 for a 5-μL marble, the liquid marbles were assumed to be spherical in our experiments^12^. We also assumed that the marbles maintain the spherical shape during the motion. Although the marble is spherical in shape, its physical properties differ from that of a solid spherical particle. For a solid spherical particle, the contact angle is constant regardless of floating position due to its rigidity. The meniscus angle can hence be calculated if the contact angle and surface inclination at the TPCL are known. For a floating marble, the apparent contact angle changes due to the deformation of the marble itself^15^. In this paper, the deformation of the floating marble is negligible due to the small volume used. Therefore, the model is similar to that of a solid spherical particle. For larger floating marbles (>10 μL), this deformation needs to be taken into account.

Capillary force arising from the edge of the container is neglected, as the meniscus created by the container is miniscule and far away from the floating marbles. The marble is assumed to maintain its initial volume throughout the experiment as the time scale of the experiments (~30 seconds from sample preparation until the end of collision) is much smaller than that of the evaporation process of the marble of approximately one hour.

## Materials and Methods

A 5 m (mole/kg) NaCl stock solution was prepared and diluted to desired concentrations (NaCl acquired from Chem-supply, dissolved in DI water). NaCl solutions of 0 to 5 m with 1 m interval were used as carrier liquids and liquids of the marble. Magnetite (Sigma-Aldrich^®^ iron oxide (II, III) 5 μm nominal diameter) was mixed with the NaCl solutions at 0.25 wt%. Weight measurements were conducted using an electronic balance (RadWag^®^ AS82/220.R2 analytical balance). The mixture was shaken thoroughly to increase particle suspension before being dispensed onto a bed of polytetrafluoroethylene (PTFE) powder (Sigma-Aldrich^®^ 1 μm nominal diameter, *ρ* = 2.2 g/cm^3^), as reported previously^32^. A 5-μL droplet was dispensed using a micropipette (Thermo Scientific Finnpipette 4500 0.5–10 μL) to ensure high accuracy. The micropipette has an uncertainty of ±4.3%. The liquid marble was formed by rolling the NaCl solution and magnetite mixture droplet in the powder bed, followed by rolling it around on a clean stainless steel spoon to dislodge excess powder from the marble surface. The weight of a liquid marble was measured by averaging over 10 marbles^36^, whereas the volume of the liquid marble was calculated based on visual measurements of its diameter. The effective marble density can be found by dividing the average mass by the volume. As the floating marble has a small submerged volume and most of its weight is supported by the surface tension of the carrying liquid^15^, the change in the density of the carrier liquid has a negligible effect on the floating position.

Five experimental runs were conducted with DI water as carrier liquid and marbles containing NaCl solution (1–5 m); five runs were conducted with NaCl solution as carrier liquid (1–5 m) and marbles containing DI water; one run was conducted with DI water as both carrier liquid and liquid marble content. Each run was repeated three times. All experimental works were carried out at a temperature of 293.5 ± 0.5 K and atmospheric pressure.

**Figure 2:**
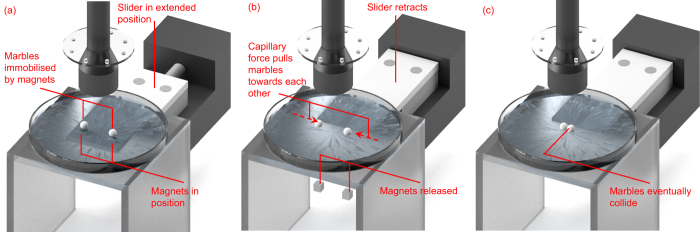
Experimental setup for the measurement of friction correction factor *β* (not drawn to scale). The Petri dish has a transparent base to show the permanent magnets and sliding platform underneath. We used an opaque white base in all of experiments to facilitate image processing. (**a**) Floating marbles containing magnetite held in their initial positions by magnets underneath. The magnets rest on a slider, which is in its extended position. (**b**) The slider retracts towards the motor, dropping the permanent magnets. This releases the floating marbles that are then free to move under capillary force. Image recording starts. (**c**) The marbles eventually collide and the recording stops.

A 140-mm-diameter Petri dish with an opaque white base was centred on a stationary platform with two square-shaped through holes spaced 15 mm apart. The Petri dish diameter is 140 mm and made of polystyrene, which has a contact angle of about 90° with water^37^. Two identical, small, cubic permanent magnets (4.5 mm for all edges) were placed inside the through holes with the same poles facing up. The characterisation data of these magnets are provided in the supplementary information. These magnets are resting on a separate sliding platform mounted on a linear stage motor (Zaber Technologies T-LS28M). All platforms were laser-machined poly(methyl methacrylate) (PMMA) slabs. The petri dish was filled with water up to a depth of about 5 mm. A USB camera (Ximea MQ013CG-ON with 0.3X EO telecentric lens) was mounted directly above the centre of the Petri dish, as shown in Fig. 2. The two marbles were placed on the liquid surface in the vicinity of the permanent magnets, one after another. The floating marbles were attracted to the permanent magnets and were then immobilised. Once the marbles were in place, the linear stage was triggered to withdraw the platform supporting the permanent magnets at a speed of 4 mm/s. When fully withdrawn, the permanent magnets were dropped and video recording was initiated immediately. As the magnets were about 4.5 mm away from the floating liquid marbles, the effective magnetic flux density is about 0.02 T, too small to magnetise the magnetite particles. Thus, magnetic force is negligible in the collision process. The entire path of collision of the marbles was recorded within less than 5 seconds. Video recording was conducted at a resolution of 638 × 508 pixels and a fixed frame rate of 50 frames per second (fps). The video was then processed frame-by-frame using MATLAB (MathWorks) to extract the centroid position of individual marbles. The data points were then fitted with a curve and the best fit generated the value of *β*.

## Results and Discussion

When the marbles were floating above the magnets, they experienced vertical forces from the magnets. Hence the floating position would be lower with a larger contact radius. Once the magnets were dropped, the liquid marbles rapidly recover their stable positions without any influence of the magnetic field. Therefore neither the magnets nor the magnetite play any role in the subsequent marble motion generated by the capillary force. Video recordings show that marble pairs collided and bounced off each other elastically. Some runs with 5 m NaCl marbles collided with enough impact to rupture one of the marbles. A sample video recording is provided in the supplementary information.

**Figure 3:**
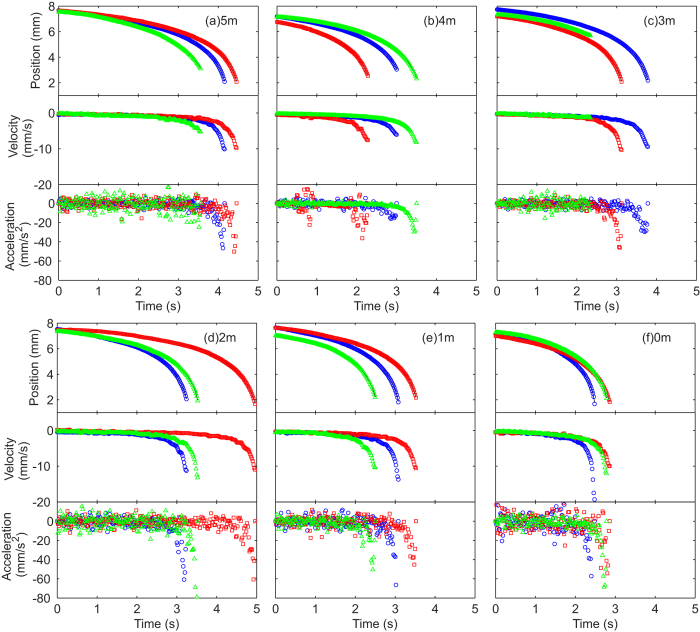
Position, velocity and acceleration versus time of DI water marbles floating on carrier liquids with different NaCl concentrations (in molals or moles/kg). The different colours and shapes represent different runs. Solid lines show fitting curves using smoothing spline. All the graphs share the same scales.

**Figure 4:**
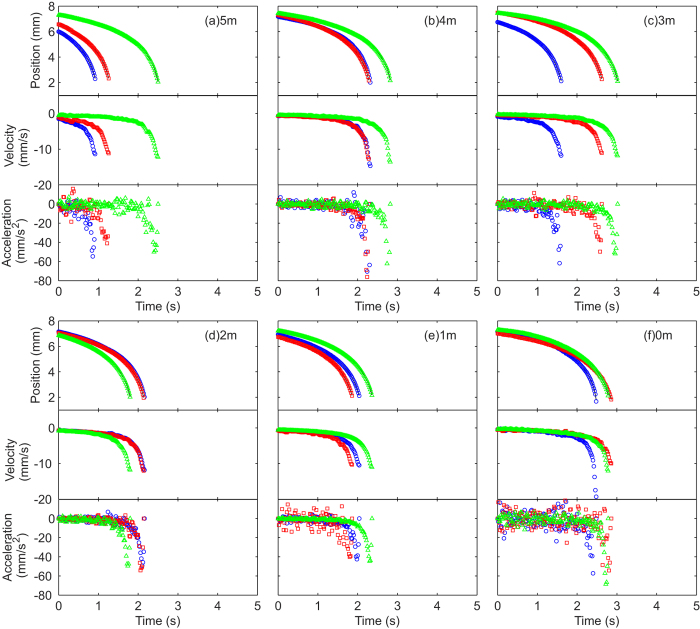
Position, velocity and acceleration versus time for marbles containing NaCl solution with different concentrations (in molals or moles/kg) floating on DI water. The different colours and shapes represent different runs. Solid lines show fitted curves using smoothing spline. All the graphs share the same scales.

Table 1 shows effective densities *ρ*_*m*_ of the marble needed to solve for Equation (9). By solving this equation, the position and velocity for different NaCl concentrations can be determined, Figs 3 and 4. Note that the curves do not overlap for the same concentration due to the slightly different initial positions and velocities.

Based on the measured initial values, a curve (solid line) is fitted for each experimental run using different values of *β*. Although NaCl can be dissolved up to 6 m, solid precipitation was observed after extended period of time. Therefore, 5 m was the maximum concentration attempted. NaCl solution was used because: (i) NaCl salt is cheap and readily available; (ii) Properties of NaCl solution are well understood and widely reported; (iii) NaCl has high solubility in water; (iv) NaCl is one of the few additives which increases the viscosity and surface tension of water, hence reducing errors generated by ambient air currents. At 5 m, the surface tension, the density and the dynamic viscosity of NaCl solution are 80.0 mN/m^38^, 1.169 g/cm^3^ 39, and 1.71 mPa∙s^40^, respectively. All the intermediate values were acquired from the respective references. Although NaCl solution is significantly more viscous than water, the increases in surface tension and density are minor.

Figures 3 and 4 show the experimental data of marble position *x*. A smoothing spline was fitted to the data points to reduce data spread caused by centroid calculations in the image analysis process. The smoothed curve was differentiated with respect to time to yield the velocity data; and differentiated twice to yield the acceleration data. As the smoothing magnitude was relatively small, minute spreads in the position data were amplified in the acceleration data.

**Figure 5:**
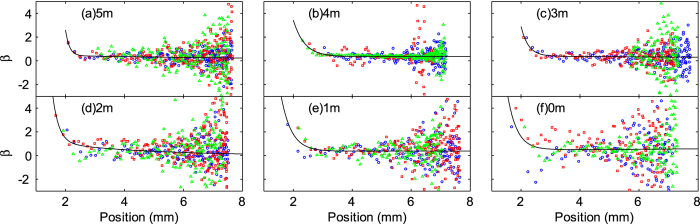
Correction factor *β* versus position of DI water marbles floating on carrier liquids with different NaCl concentrations (in molals or moles/kg). Different colours and shapes represent different runs for each concentration.

**Figure 6:**
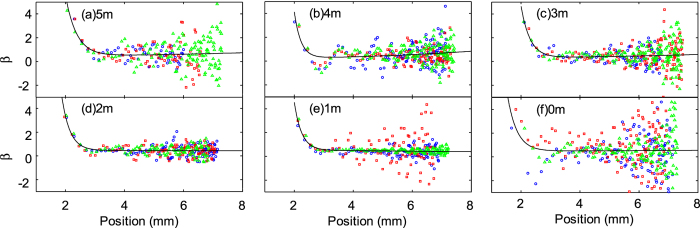
Correction factor *β* versus position for marbles containing NaCl solution with different concentrations (in molals or moles/kg) floating on DI water. Different colours and shapes represent different runs for each concentration.

**Figure 7:**
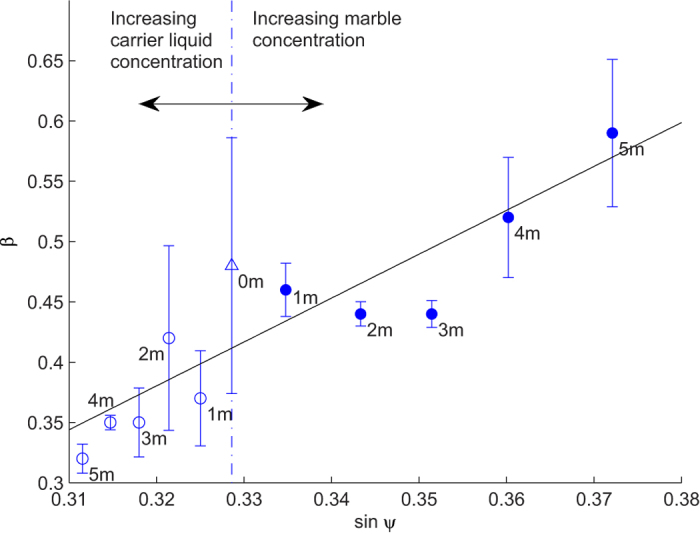
The friction correction factor *β* versus the sine function of the meniscus angle sin *ψ*. The solid black line shows the linear relationship between *β* and sin *ψ*. Data points of *β* of water marbles floating on NaCl solution are located at the region to the left of the vertical reference line (empty circles). The respective NaCl concentration of the carrier liquid is shown next to the data point. Data points of *β* of NaCl solution marbles floating on water are located at the region to the right of the vertical reference (solid circles). The respective NaCl concentration of the marble is shown next to the data points. Errors indicated were derived from different *β* values corresponding to individual experimental runs.

Figures 5 and 6 show the calculated *β* values from each data point, plotted against the marble position. *β* values are relatively constant for a large distance (*x* > 3 mm) but increases rapidly as the distance shortens. Thus, a two-term exponential fitting function was fitted onto the data points to obtain an averaged *β*:


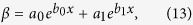


where *a* and *b* are fitting parameters. Ideally, the correction factor should be independent of the position, assuming that the floating body is a rigid sphere. However, this assumption might not be true as the correction factor is a function of the position, Figs 5 and 6. The submerged portion of the floating marble might deform, especially at higher speeds or at lower position values, affecting the value of the correction factor. The proposed exponential fitting equation was only used as an empirical description of the obtained data. This function was chosen because it takes into account the two distinct regions of *β* values. The data spread increases with increasing distance. This uncertainty is likely caused by the fact that the marbles dwelled at the vicinity of the starting position (≈7.5 mm) with low velocities (<1 mm/s). Thus, they were very susceptible to micro currents and minute vibration sources. Since *β* is relatively linear in the region of (3 mm < *x* < 7.5 mm), we took the average value of *β* at these two position as the correction factor for the coefficient of friction 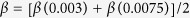. Figure 7 shows the averaged value of *β* versus the sine function of the meniscus angle 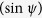.

If the marble floats with its equator intersecting the interface, its meniscus angle *ψ* is zero. Consequently, the value of *β* should be 0.5. In our case, the liquid marble floats relatively high on the free surface with a small meniscus angle *ψ*. Therefore, we expect a smaller correction factor of *β *< 0.5. Our results disagrees with this hypothesis to a certain extent, as experiments of water marble floating on water have an average *β* of 0.52, higher than the expected value. This discrepancy is likely contributed by the perturbation of the water surface due to the weight of the marble and rough contact area caused by the particle coating. Nevertheless this value is very close to our estimated value reported earlier^13^.

By increasing the surface tension of the carrier liquid, the marble is lifted higher with a smaller meniscus angle *ψ*. Hence, the correction factor *β* is reduced (Fig. 7, region to the left of the dash-dot line). Conversely, increasing the density of the marble generates a larger *ψ*, as *β* is increased (Fig. 7, region to the right of blue dash-dot line). The value of *β* may exceed 0.5 because increasing both the submerged area and meniscus angle contribute to a larger flow resistance. The carrier liquid surface tensions and marble effective densities were adjusted to reflect the changes of the meniscus angle, with reference to Equation (12). Within our experimental dataset, *β* can be approximated to have a linear relationship with sin *ψ* where 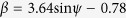, *r*^2^ = 0.73. The fitting function is shown as a solid line in Fig. 7. With this fitting function, we can estimate *β* of a small floating marble based on the marble and carrier liquid properties. Substituting Equation (12) into our fitted line results in the approximated relationship:


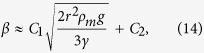


where C_1_ and C_2_ are constants (C_1_ = 3.64 and C_2_ = −0.78 in our case). Equation (14) provides a convenient way to estimate *β* for small floating marbles without the need to run an experiment for every parameter change.

This model relating the correction factor *β* to the meniscus angle *ψ* is limited for small, floating and non-wetting spherical objects because the buoyancy forces are relatively small. Since this model assumes spherical marbles, it is valid for small Bond numbers 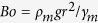 where *γ*_*m*_ is the marble effective surface tension. For future works, using compatible liquids of larger differences in surface tension and density can further expand the range of the meniscus angle ψ.

## Conclusion

This paper reports the experimental determination of the Stokes friction correction factor of a small floating liquid marble for a small range of meniscus angles. The experimental setup was designed to simplify the calculations of the various force terms involved in the marble motion generated by capillary force. We exploited the non-wetting properties of a liquid marble and its magnetic actuation to design the experiments. The correction factor of a 5-μL water marble was found to be about 0.52. We found that the correction factor could be estimated to vary linearly with the sine function of the meniscus angle, which itself could be expressed in terms of marble size, effective density, and carrier liquid surface tension. The method reported here provides a convenient way to estimate the correction factor of a small floating liquid marble without the need of characterising its motion. The known friction factor enables proper designing and modelling liquid marbles as a digital microfluidics platform for various applications.

## Additional Information

**How to cite this article**: Ooi, C. H. *et al*. Measuring the Coefficient of Friction of a Small Floating Liquid Marble. *Sci. Rep.* **6**, 38346; doi: 10.1038/srep38346 (2016).

**Publisher's note:** Springer Nature remains neutral with regard to jurisdictional claims in published maps and institutional affiliations.

## Supplementary Material

Supplementary Information

Supplementary Video

## Figures and Tables

**Table 1:** Mean effective density of the marble for different NaCl concentrations.

NaCl Concentration (m)	Mean marble effective density (g/cm^3^)	Standard deviation
0	0.958	0.193
1	1.129	0.162
2	1.120	0.080
3	1.137	0.115
4	1.159	0.105
5	1.246	0.125

The density values are calculated based on marble mass and volume measurements.
