# Supplementary material for: Measuring the Coefficient of Friction of a Small Floating Liquid Marble
Source: Sci Rep. 2016 Dec 2;6:38346. doi: 10.1038/srep38346 (PMC5133567; doi:10.1038/srep38346)
Supplement: Supplementary Information [file srep38346-s1.pdf]

Measuring the Coefficient of Friction of a Small Floating Liquid Marble

Supplementary Information

Chin Hong Ooi,[a] Anh Van Nguyen,[b] Geoffrey M. Evans,[c] Dzung Viet Dao,[a] Nam-Trung Nguyen*[a]

*E-mail: nam-trung.nguyen@griffith.edu.au

a. Queensland Micro- and Nanotechnology Centre, Griffith University, 170 Kessels Road, 4111 Queensland, Australia.
b. School of Chemical Engineering, University of Queensland, St Lucia, 4072 Queensland, Australia.
c. School of Engineering, University of Newcastle, Callaghan, NSW 2308, Australia.

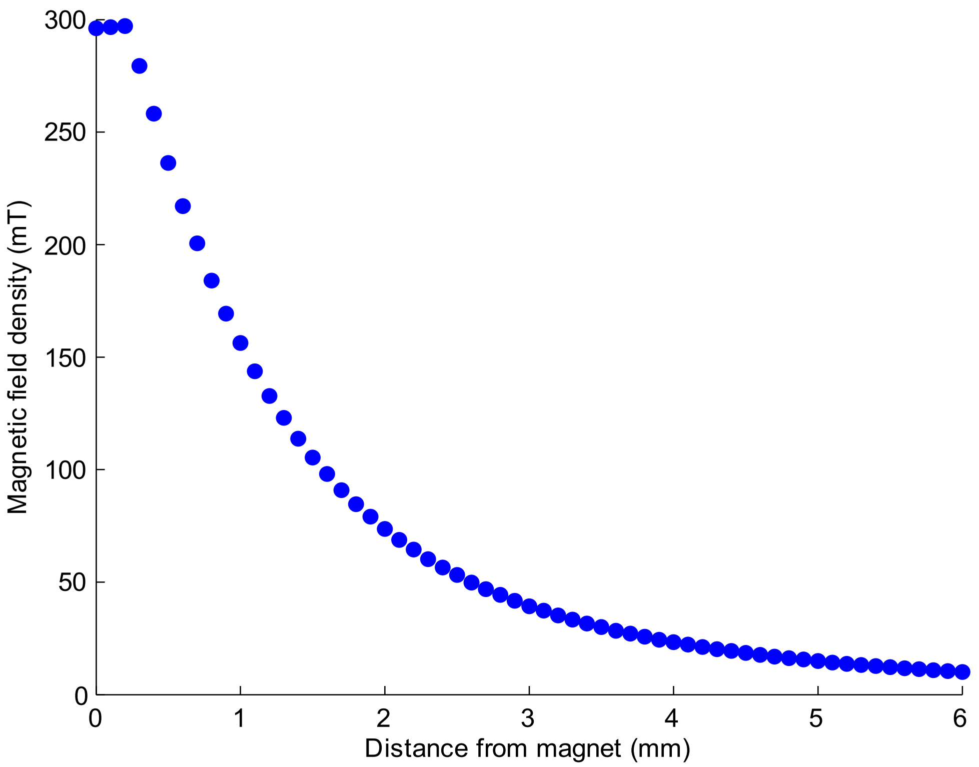

S1 Characterisation of permanent magnets. The magnetic field density of the square magnets used was measured using a Gaussmeter and plotted against the distance from the magnet. In our case, the magnets were placed approximately 4.5 mm away from the floating marbles, yielding about 20 mT.

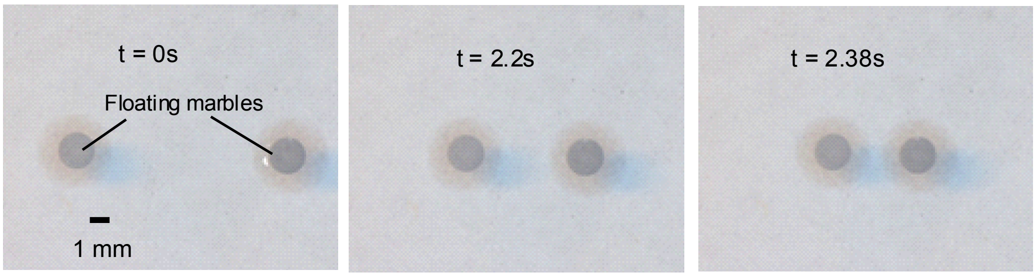

S2 Images from the sample video of the top view of a colliding floating marble pair. The marbles used here has a NaCl concentration of 1 mole/kg floating on water. t = 0s indicates the moment the magnets were released. The marbles have a greyish-black appearance due to the dispersed magnetite powder inside the marbles. This is the reason for using a white background. The faint halo surrounding the marbles are shadows caused by the LED ring lights. Images were taken at 20 fps.

S3 Sample video of the top view of a colliding floating marble pair. The marbles used here has a NaCl concentration of 1 mole/kg floating on water.
